# Prevalence and genotype distribution of human papillomavirus in Czech non-vaccinated heterosexual couples

**DOI:** 10.1186/s12985-021-01551-x

**Published:** 2021-04-15

**Authors:** Hana Jaworek, Vladimira Koudelakova, Ivana Oborna, Blazena Zborilova, Jana Brezinova, Dagmar Ruzickova, Jana Vrbkova, Pavla Kourilova, Marian Hajduch

**Affiliations:** 1 Institute of Molecular and Translational Medicine, Faculty of Medicine and Dentistry, Palacky University Olomouc, Hnevotinska 1333/5, 779 00 Olomouc, Czech Republic; 2 Fertimed Ltd., Boleslavova 2, 776 00 Olomouc, Czech Republic; 3 SpermBank International, Katerinska 13, 779 00 Olomouc, Czech Republic; 4 Arleta IVF Ltd., Komenskeho 702, 517 41 Kostelec nad Orlici, Czech Republic

**Keywords:** Human papillomavirus, Semen, Penile swab, Cervical swab

## Abstract

**Background:**

Data about the genotype-specific human papillomavirus (HPV) prevalence in the Czech Republic is limited. We aimed to evaluate the prevalence and concordance of genotype-specific HPV infection detected in semen samples, penile swabs and cervical swabs from non-vaccinated heterosexual couples without HPV-associated disease.

**Methods:**

Semen samples and penile swabs were collected from male partners and cervical swabs were collected from female partners of heterosexual couples treated for infertility (n = 195). Presence of HPV DNA in semen samples and cervical swabs was analyzed using the cobas^*®*^ HPV Test and PapilloCheck^*®*^. Only the PapilloCheck^*®*^ test was used to detect HPV in penile swabs. The genotype-specific prevalence and concordance of HPV infection not targeted by vaccine were evaluated using Fisher exact test.

**Results:**

Both partners were infected with any HPV type in 13.8% (27/195) of couples and, of these couples, 55.6% (15/27) harbored at least one mutual genotype. High-risk HPV (hrHPV) genotypes were detected in 12.3% (24/195) of semen samples, 31.3% (61/195) of penile swabs, and 19.5% (38/195) of cervical swabs (*P* < 0.001). The most prevalent hrHPV genotype were HPV53 (2.56%; 5/195) in semen samples, HPV16 (6.67%, 13/195) in penile swabs and HPV39 (3.59%, 7/195) in cervical swabs. Low-risk (lrHPV) genotypes were detected in 5.13% (10/195) of semen samples, 15.9% (31/195) of penile swabs, and 4.10% (8/195) of cervical swabs (*P* < 0.001). Male sexual partners of HPV-positive women were more likely to be infected with at least one of the same HPV types than female sexual partners of HPV-positive men (34.9% vs. 17.9%, *P* = 0.055).

**Conclusions:**

This study showed that the detection of HPV infection differ by anatomic site and gender. Regardless the anatomic site, high prevalence of HPV genital infection was found in both Czech men and women.

**Supplementary Information:**

The online version contains supplementary material available at 10.1186/s12985-021-01551-x.

## Background

Human papillomaviruses (HPVs) are agents of common sexually-transmitted diseases that affect sexually-active human populations [1]. Although most HPV infections are asymptomatic and transient [2, 3], persistent infection could lead to development of benign (low-risk HPV [lrHPV] types) or malignant (high-risk HPV [hrHPV] types) lesions [1]. Nearly all cervical cancer cases are caused by hrHPVs. Moreover, hrHPVs are also linked to development of precancerous and cancerous lesions in other anatomical sites in both males and females, such as the vagina, vulva, anus, penis and oropharynx [4]. Anogenital warts and recurrent respiratory papillomatosis are caused by lrHPVs [1].

In the Czech Republic, data on the genotype-specific prevalence of HPV in women is limited [5–8]. Even less is known about HPV prevalence in the Czech male population [9, 10]. HPV genotyping is attractive for use in cervical cancer screening because it offers the possibility for integrating HPV screening with HPV vaccination monitoring. HPV vaccination of girls aged 13–14 years has been funded since 2012 in the Czech Republic and was extended to boys in 2018 [11]. Evidence of HPV prevalence and concordance in couples is important for evaluating HPV vaccine impact and for monitoring the spread of specific HPV types before and after introducing a vaccine to a population. Therefore, we aimed to evaluate the prevalence and concordance of genotype-specific HPV infection in semen samples, penile swabs and cervical swabs from non-vaccinated heterosexual partners.

## Methods

### Study design and inclusion criteria

**Fig. 1:**
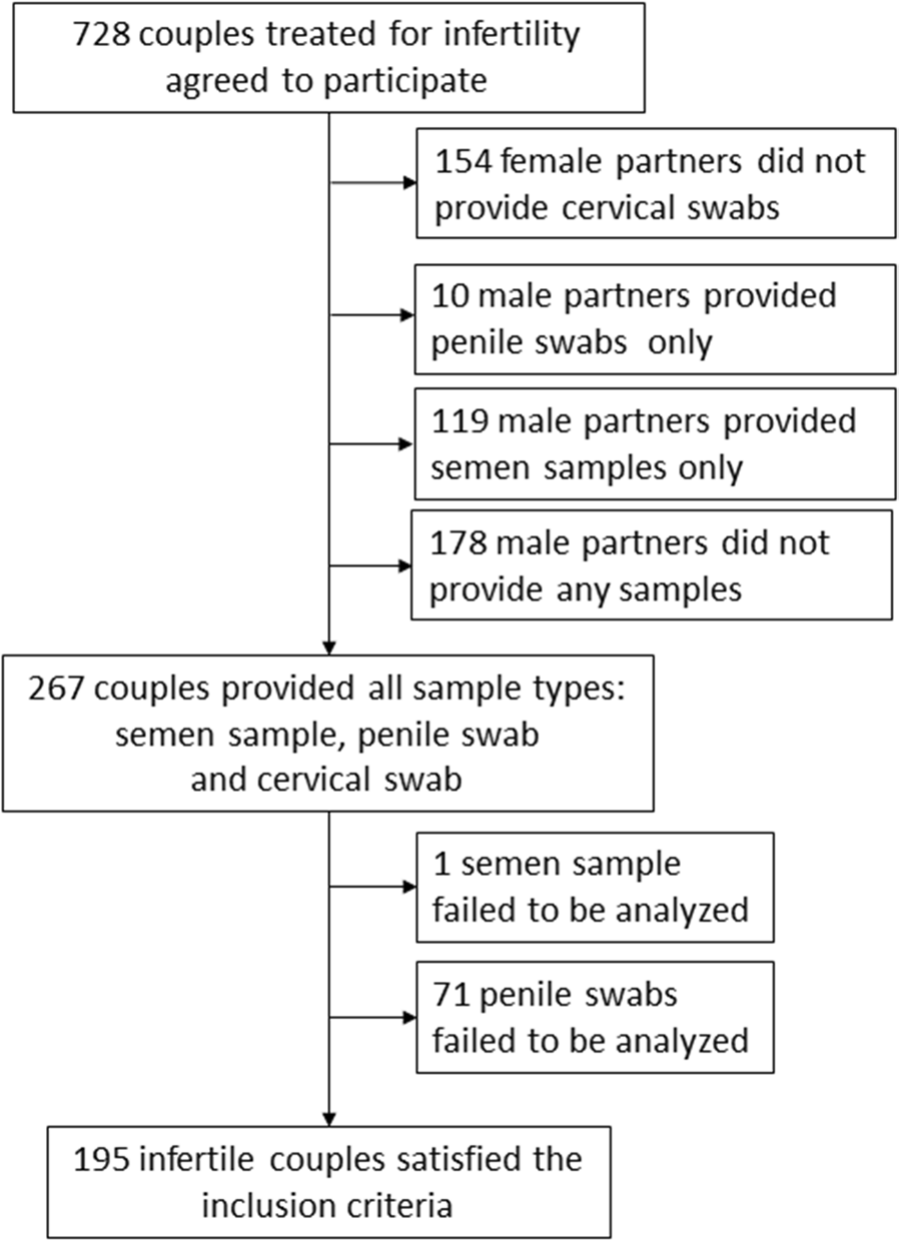
Study flowchart

Seven hundred and twenty-eight (728) couples treated for infertility in two Czech fertility centers, Fertimed Ltd., Olomouc and Arleta IVF Ltd., Kostelec nad Orlici, were enrolled into the study from July 2013 to November 2016. These two Czech fertility centers operate in the same geographical region. Long-term monogamous couples who engaged in regular sexual activity with no barrier contraception, demonstrated no symptoms of HPV-associated disease, and received no prior vaccinations against HPV were included. Only 195 couples provided both semen samples and penile swabs from male partners as well as cervical swabs from female partners that were suitable for evaluation (Fig. 1).

### Collection of samples

Male study participants self-collected penile swab samples by wiping a dry cotton swab at least three times around the coronal sulcus and the top of the glans. Each swab was then rinsed in 0.5 mL of cobas^*®*^ PCR Cell Collection Media (Roche Diagnostics GmBH, Mannheim, Germany). Semen samples were obtained at the same time by masturbation after 3–5 days of sexual abstinence. After liquefaction of the ejaculate at room temperature, at least 0.1 mL of ejaculate was put into 20 mL of cobas^*®*^ PCR Cell Collection media. Clinician-collected cervical brushes were rinsed in 20 mL of cobas^*®*^ PCR Cell Collection Media. All samples in cobas^*®*^ PCR Cell Collection Media were transported and stored at room temperature until testing according to the manufacturer’s recommendations for gynecologic samples.

### HPV DNA detection

Cervical swabs and semen samples were tested for the presence of HPV DNA using the cobas^*®*^ HPV Test (Roche Diagnostics GmBH, Mannheim, Germany) according to the manufacturer’s recommendations for cervical swabs management [12] and using the PapilloCheck^*®*^ test (Greiner Bio-One, Frickenhausen, Germany) [13] as described previously [5]. DNA from penile swabs was isolated by QIAamp^*®*^ DNA Micro kit (Qiagen, Hilden, Germany) and eluted with 40 μL of diethyl pyrocarbonate (DEPC) treated water. Isolated DNA was then tested using PapilloCheck^*®*^ [13]. Samples with HPV detection failure were repeated three times. HPV detection repeatedly failed in 71 penile swab samples and 1 semen sample due to insufficient amount of genetic material. Participants with failed samples were excluded from the study.

**Fig. 2:**
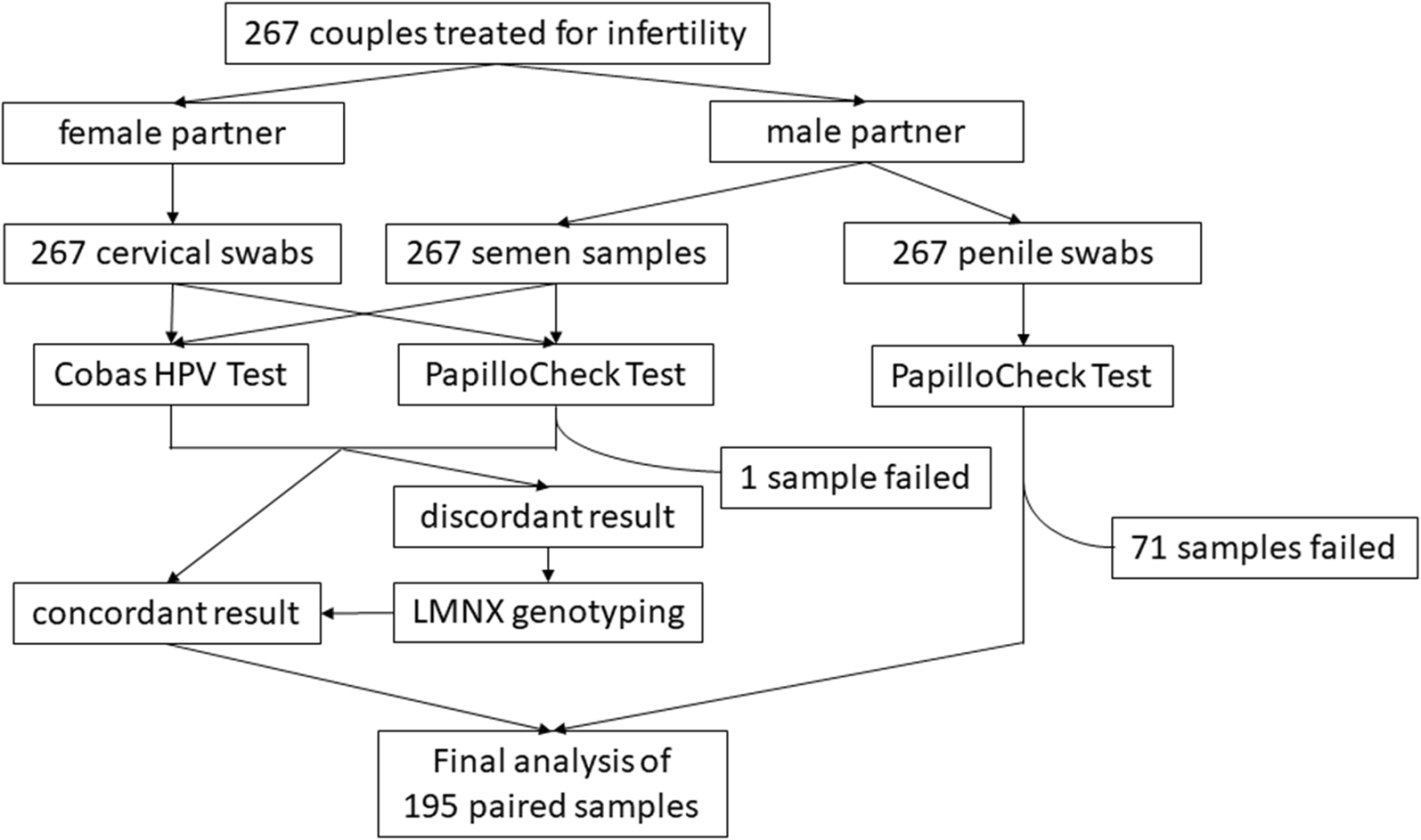
Methods used for HPV detection in different sample types

The cobas^*®*^ HPV Test and the PapilloCheck^*®*^ test gave discordant results for 8 out of 195 cervical swabs and 4 out of 195 semen samples included in the study. These 12 samples were additionally tested using the LMNX Genotyping Kit HPV GP (Diassay, Rijswijk, The Netherlands) [14] as described previously [5]. The final HPV result for a given cervical swab or semen sample was determined by concordant results of at least two HPV detection methods, as described previously [6]. The HPV result for a given penile swab was based only on the PapilloCheck^*®*^ test (Fig. 2).

We examined any-type concordance (both partners are HPV positive), same-type concordance (both partners are HPV positive for 1 or more mutual HPV types), and type-specific concordance for vaccine-targeted genotypes HPV16, 18, 31, 33, 45, 52, 58, 6, and 11. For this type of analysis, men was considered as HPV positive when either the penile swab or the semen sample was HPV positive.

### Statistical analysis

Statistical analysis was performed using R, ver. 3.5.2 (Pearson chi-square test, Fisher exact test or Wilcoxon exact test, as appropriate). A *P*-value ≤ 0.05 was considered statistically significant.

## Results

### HPV positivity rates

DNA of at least one of 18 hrHPV genotypes or 6 lrHPV genotypes was detected in 15.9% (31/195) of semen samples, 41.0% (80/195) of penile swabs, and 22.1% (43/195) of cervical swabs (*P* < 0.001; Fisher exact test). At least one partner was positive for hrHPV or lrHPV in 51.3% (100/195) of couples. High risk HPV genotypes were detected in 12.3% (24/195) of semen samples, 31.3% (61/195) of penile swabs, and 19.5% (38/195) of cervical swabs (*P* < 0.001; Fisher exact test). Low risk HPV genotypes were detected in 5.13% (10/195) of semen samples, 15.9% (31/195) of penile swabs, and 4.10% (8/195) of cervical swabs (*P* < 0.001; Fisher exact test; Table 1).

**Table 1:** Prevalence of hrHPV and lrHPV genotypes detected in couples

	Semen sample (n = 195)	Penile swab (n = 195)	Cervical swab (n = 195)	Fisher exact test
	n (%)	n (%)	n (%)	***P***
HPV16	4 (2.05)	13 (6.67)	6 (3.08)	0.066
HPV18	0 (0)	1 (0.51)	0 (0)	1
HPV31	2 (1.03)	3 (1.54)	2 (1.03)	1
HPV33	0 (0)	1 (0.51)	3 (1.54)	0.331
HPV35	0 (0)	1 (0.51)	0 (0)	1
HPV39	1 (0.51)	7 (3.59)	7 (3.59)	0.068
HPV45	1 (0.51)	0 (0)	2 (1.03)	0.777
HPV51	4 (2.05)	8 (4.10)	2 (1.03)	0.160
HPV52	2 (1.03)	3 (1.54)	6 (3.08)	0.406
HPV53	5 (2.56)	8 (4.10)	4 (2.05)	0.554
HPV56	3 (1.54)	8 (4.10)	3 (1.54)	0.198
HPV58	1 (0.51)	1 (0.51)	3 (1.54)	0.627
HPV59	0 (0)	5 (2.56)	2 (1.03)	0.076
HPV66	2 (1.03)	6 (3.08)	0 (0)	0.032
HPV68	2 (1.03)	4 (2.05)	2 (1.03)	0.740
HPV70	0 (0)	5 (2.56)	4 (2.05)	0.086
HPV73	0 (0)	0 (0)	2 (1.03)	0.332
HPV82	1 (0.51)	3 (1.54)	1 (0.51)	0.627
HPV6	1 (0.51)	5 (2.56)	0 (0)	0.052
HPV11	0 (0)	0 (0)	0 (0)	1
HPV40	2 (1.03)	3 (1.54)	0 (0)	0.38
HPV42	2 (1.03)	13 (6.67)	4 (2.05)	**0.006**
HPV43	1 (0.51)	4 (2.05)	1 (0.51)	0.38
HPV44/55	5 (2.56)	14 (7.18)	3 (1.54)	**0.010**
Other HPV	1 (0.51)	0 (0)	0 (0)	1
lrHPV	10 (5.13)	31 (15.9)	8 (4.10)	< **0.001**
hrHPV	24 (12.3)	61 (31.3)	38 (19.5)	< **0.001**
Total	31 (15.9)	80 (41.0)	43 (22.1)	< **0.001**

Statistically significant data (*P* ≤ 0.05) are shown in bold

High-risk HPV (hrHPV) includes HPV31, 33, 35, 39, 45, 51, 52, 53, 56, 58, 59, 66, 68, 70, 73, and 82 genotypes

Low-risk HPV (lrHPV) includes HPV6, 11, 40, 42, 43, 44/55

The hrHPV genotypes most frequently detected in men were HPV53 (2.56%; 5/195) in semen samples and HPV16 (6.67%, 13/195) in penile swabs. In cervical swabs, HPV39 was the most frequently identified hrHPV genotype (3.59%, 7/195). The lrHPV genotype most frequently detected in semen samples (2.56%; 5/195) and penile swabs (7.18%; 14/195) was HPV44/55 whereas HPV42 was the most frequently identified lrHPV genotype in cervical swabs (2.05%; 4/195). HPV42 and HPV 44/55 were detected significantly more frequently in penile swabs than in semen samples or cervical swabs (6.67% vs. 1.03% vs. 2.05%, *P* = 0.006; 7.18% vs. 2.56% vs. 1.54%, *P* = 0.010; Fisher exact test). No other significant differences in genotype-specific occurrence were found between the sample types compared (Table 1).

Among HPV positive participants, HPV single-type infection and co-infection was detected in 74.2% (23/31) and 25.8% (8/31) of semen samples; 72.5% (58/80) and 27.5% (22/80) of penile swabs and 76.7% (33/43) and 23.3% (10/43) of cervical swabs (Additional file 1).

### HPV concordance

The concordance in positive testing for any HPV type was significantly higher in paired samples of semen samples and penile swabs (0.321) than in penile swabs and cervical swabs (0.281) or in semen samples and cervical swabs (0.072; *P* < 0.001; Fisher exact test). The same trend was observed for concordance comparisons of the presence of 1 or more of the same HPV genotypes in paired samples (0.261 vs. 0.139 vs. 0.014, *P* < 0.001; Fisher exact test) (Additional file 2).

Then we compared HPV positivity concordance between partners. Both partners were positive for any type of HPV in 13.8% (27/195) of couples (Additional file 2). One or more of the same HPV genotypes were detected in 7.69% (15/195) of couples. Same-genotype concordance for people with HPV-infected partners was higher for men than for women. Among male partners of HPV-positive women, 34.9% (15/43) were infected with 1 or more of the same HPV genotype. In contrast, among female partners of HPV-positive men, 17.9% (15/84) were infected with 1 or more of the same HPV genotype (*P* = 0.055; Fisher exact test) (Additional file 2).

Both partners of only 4 couples tested positive for bivalent vaccine-targeted genotypes (HPV16) as well as for quadrivalent vaccine targeted genotypes (HPV16). HPV genotypes targeted by nonavalent vaccines were detected in both partners of 8 couples (HPV16, HPV31, HPV33 and HPV52).

### HPV positive detection and age

The median ages of male partners and female partners from couples (n = 195) were 35 years (range, 22–57 years) and 32 years (range 20–46 years, *P* < 0.00; Wilcoxon exact test). The median age of participating men presenting hrHPV-positive semen samples was no different from that of men presenting hrHPV-negative semen samples [median age, 35 years (range 22–48 years) vs. 35 years [range 24–57 years], respectively, *P* = 0.669; Wilcoxon exact test]. Similarly, the median age of participating men presenting hrHPV-positive penile swabs was no different from that of men presenting hrHPV-negative penile swabs [median age, 36 years (range 23–51 years) vs. 35 years (range 22–57 years), respectively, *P* = 0.656; Wilcoxon exact test]. Likewise, for participating women, no difference in median age was observed between those presenting hrHPV-positive cervical swabs and those presenting hrHPV-negative cervical swabs [median age, 32 years (range 22–44 years) vs. 32 years (range 20–46 years), respectively, *P* = 0.367; Wilcoxon exact test].

Moreover, no association was identified between age and hrHPV, lrHPV or HPV-positive detection in general for any type of tested sample.

## Discussion

We evaluated the prevalence and concordance of genotype-specific HPV infection in semen samples and penile and cervical swabs from 195 non-HPV-vaccinated heterosexual Czech couples treated for infertility.

Our findings showed that both partners were infected with any HPV type in 13.8% of couples and 15/27 (55.6%; 95% CI = 35.3–74.5%) of these couples harbored at least one mutual genotype. Contrary to our findings meta-analysis conducted by Reiter et al. [15] reports more than two times higher occurrence of HPV infection in both sexual partners (37.7%; 975/2972) than the occurrence we observed. The discrepancy could be caused by Reiter’s et al. [15] predominant inclusion of studies focused on HPV occurrence in individuals with one sexual partner having HPV-associated disease. Persistent HPV infection and high viral load increase the risk of virus transmission to the sexual partner. However, genotype-specific concordance of at least one genotype reported in the meta-analysis was 63.2% (95% CI = 49.1–75.3%) [15], thus corresponding the findings of our study. This level of concordance is consistent with high HPV transmissibility, estimated at about 40% per unprotected sexual act [15, 16].

We found that male sexual partners of HPV-positive women are more likely to be infected with at least one same HPV genotype than female sexual partners of HPV-positive men (34.9% vs. 17.9%, *P* = 0.055; Pearson chi-squared test). This finding aligns with studies suggesting more likely genital transmission of α-HPV from women to men [17–19]. Other studies indicate that men acquire more transient genital HPV infections than women at almost any age, whereas HPV antibody levels are much lower in men throughout their lives [20, 21]. In general, the prevalence of genital HPV infection in men is higher than in women [1, 22]. Genital HPV infection is also less likely to persist [20, 23] and tends to vary less by age [20, 24] in men than in women. We found no association between age and hrHPV, lrHPV or HPV-positive detection in general for any type of tested sample. Nevertheless, this could be caused by small number of cases in border age categories, various representation in individual age categories or relatively small numbers of HPV positive cases.

We found that HPV infection in penile swabs was detected two times more frequently than in cervical swabs (41.0% vs. 22.1%), and the frequency of detection in semen samples was even lower (15.9%). Similar to our study, Giuliano et al. [25] reported that genital HPV in men occurs most frequently at the penis and least frequently at the urethra or semen samples. The proportion of hrHPV and lrHPV-positive detection in women appeared similar (hrHPV 14–15%; lrHPV 18%) in Giuliano et al. [25], however the prevalence of lrHPV (39%) seemed higher than that of hrHPV (30%) in men. In our study, hrHPV was detected more frequently than lrHPV in both penile (31.3% vs. 15.9%, *P* < 0.001; Pearson chi-squared test) and cervical swabs (19.5% vs. 4.10%, *P* < 0.001; Pearson chi-squared test). This difference could be caused by regional and age variabilities in studied populations.

The global HPV prevalence in women with normal cytology findings is estimated at 11.7% with considerable regional differences. Regardless of the region, the highest HPV prevalence is observed in women under 25 years of age (24.0%) and declines with age with up to 7.5% HPV prevalence observed in women older than 55 years [1, 24]. Only 5.6% (11/195) of women enrolled in our study were younger than 25 years of age, yet HPV prevalence was 22.1% and hrHPV prevalence was 19.5%. However, other Czech study report that hrHPV prevalence in cytologically negative women is 15.6% (203/1,302) [7] and HPV prevalence in women treated for infertility is usually even higher [26, 27]. In general, the most frequently detected hrHPV genotype in asymptomatic women is HPV16 (3.2%), followed by HPV18 (1.4%) and HPV52 (0.9%) [1, 24]. In our study, HPV39 (3.59%) was the most frequently detected type, followed by HPV16 (3.08%) and HPV52 (3.08%). Genotype prevalence in our study could be affected by limited number of included women.

Contrary to observations in women, HPV prevalence data in the asymptomatic male population are very limited and no published study reports on HPV prevalence in the Czech male population is available to date. HPV is rarely tested in men, with testing usually occurring only because of HPV-associated disease, or due to research mainly on the male partners of women with HPV-associated disease.

The largest study focused on genital HPV prevalence in healthy men was HIM study [22] which was conducted among men from Brazil, Mexico and the United States of America and included penile swabs of 1,160 participants. In this study, hrHPV infection was detected in 29.7% samples and the most commonly detected hrHPV genotypes were HPV16 (6.5%), HPV51 (5.3%), and HPV59 (5.3%). Similarly, in our study, hrHPV infection was detected in 31.3% of penile swab samples and the most commonly detected genotypes were HPV16 (6.67%), HPV51 (4.1%) and HPV56 (4.1%). Detection of lrHPV infection was observed in 38.5% of HIM study participants. Among the lrHPV, HPV84 was most commonly detected (7.7%), followed by HPV62 (7.3%) and HPV6 (6.6%) [22]. In our study, lrHPV detection was significantly lower with only 15.9% lrHPV prevalence. The most common lrHPV genotype was HPV44/55 (7.18%) and HPV42 (6.67%). The inconsistency in lrHPV prevalence between the HIM study and ours may be due to differences in HPV detection systems since our system is not able to detect HPV84 and HPV62.

In recent meta-analysis, semen samples of men from infertile couples demonstrated significantly higher HPV prevalence than semen samples from men of the general population [28]. HPV prevalence in fertility clinic attendees and men from the general population was 20.4% and 11.4% [28] with high geographical variability from 17.9 to 22.0% in fertility clinic attendees and 4.5–15.2% in men of the general population. HPV prevalence in semen samples detected in our study (15.9%) corresponds more with HPV prevalence in the general European male population (15.2%) than with HPV prevalence in European male fertility clinic attendees (17.9%). Nevertheless, the results could be biased due to the small number of HPV positive semen samples (31/195).

In the meta-analysis, hrHPV/lrHPV infection was detected in 10.0%/8.3% of men from general population and 15.5%/10.3% of male fertility clinic attendees [28]. In our study, hrHPV and lrHPV infection was detected in 12.3% (24/195) and 5.13% (10/195) of semen samples. The most common hrHPV and lrHPV types in both the general population and fertility clinic attendees were HPV16 (prevalence 4.8% and 6%) and HPV6 (prevalence 2.4% and 1.3%) [28]. In our study, HPV16 was the second most common hrHPV type (4/31; 2.05%) in semen samples, and the most common type was HPV53 (5/31; 2.56%). HPV44/55 was the most prevalent lrHPV type, with HPV6 detected only in one case. Limitation of our study is small number of included participants and therefore small numbers of HPV positive cases. Nevertheless, it is the largest study comparing HPV prevalence and HPV genotype distribution in Czech heterosexual couples.

## Conclusion

The current study draws attention to the high HPV prevalence not only among Czech women but also in Czech men and shows the differences in HPV prevalence and genotype-specific concordance by anatomic sampling sites and gender in Czech heterosexual couples.

## Supplementary Information

**Additional file 1.** Prevalence, single-type infection and coinfection of hrHPV and lrHPV genotypes detected in participating couples. Statistically significant data (*P *≤ 0.05) are shown in bold. HrHPV includes HPV31, 33, 35, 39, 45, 51, 52, 53, 56, 58, 59, 66, 68, 70, 73, and 82 genotypes. LrHPV includes HPV6, 11, 40, 42, 43, 44/55. The HPV result for a given sample was obtained when at least two detection methods were concordant. The presence of HPV53, 70, 73, 82 and lrHPV genotypes (HPV6, 11, 40, 42, 43, 44/55) was evaluated using PapilloCheck^®^ HPV-Screening only. The presence of HPV in penile swabs was evaluated using PapilloCheck^®^ HPV-Screening only.

**Additional file 2.** HPV concordance in paired samples taken from male and female partners of participating couples. *CS* cervical swab, *PS* penile swab, *SS* semen sample, *NA* not applicable. Statistically significant data (*P*-value  < 0.05) are shown in bold. *Concordance in any type of sample from male partner with sample from female partner any-type concordance-both partners are HPV positive same-type concordance-both partners are HPV positive for 1 or more HPV types in common.

## Data Availability

The datasets used and analyzed during the current study are available from the corresponding author on reasonable request.

## Abbreviations

DEPC: Diethyl pyrocarbonate
HPV: Human papillomavirus
hrHPV: High-risk human papillomavirus
lrHPV: Low-risk human papillomavirus
